# Effects of impregnation combined heat treatment on the pyrolysis behavior of poplar wood

**DOI:** 10.1371/journal.pone.0229907

**Published:** 2020-03-17

**Authors:** Meihui Wu, Juwan Jin, Chengyang Cai, Jingbo Shi, Xuefeng Xing, Jiabin Cai

**Affiliations:** 1 Department of Wood Science, Nanjing Forestry University, Nanjing, Jiangsu, China; 2 School of Forest Sciences, University of Eastern Finland, Joensuu, North Karelia, Finland; George Mason University, UNITED STATES

## Abstract

To investigate the effects of urea-formaldehyde (UF) resin impregnation combined heat treatment (IMPG-HT) on the pyrolysis behavior of poplar wood, the chemical composition, pyrolysis characteristics, pyrolysis kinetics, and gaseous products released during pyrolysis of untreated (control), IMPG-HT, IMPG and HT woods were analyzed. The results demonstrate that IMPG-HT changes pyrolysis behavior of poplar wood significantly. Unlike the control and HT samples, the thermogravimetric / derivative thermogravimetric (TG/DTG) curves of IMPG wood shift toward lower temperature, and the shoulder on DTG curves weaken or even disappear. The maximum mass loss rate of IMPG-HT samples decreases, and carbon residual yield increases to 23% or more and activation energy (*E*) increases sharply after conversion rate (*α*) reaching 0.80. HT improves the thermal stability of IMPG wood, which is represented by the increase of decomposition temperature (*T*_*d*_) and DTG peak temperature (*T*_*peak*_) and the higher *E* value of IMPG-HT wood. For the pyrolysis gaseous products, IMPG-HT wood produces nitrogen-containing gases (HNCO and NH_3_) due to the presence of UF resin, but the amounts of these gases are less than that produced by IMPG wood because the heat treatment had removed part of *N* elements.

## Introduction

Biomass materials are widely known as potential renewable sources of energy in the future due to reduced fossil fuel resources and environmental deterioration. The reuse of wood waste has become important for energy utilization. Pyrolysis, a universally applicable method for reusing biomass materials, has gained more and more attention. The pyrolysis behaviour and mechanism of the main components of wood such as cellulose [1,2], hemicellulose [1,2], lignin [1,2,3] and extractives [2,4] as well as the pyrolysis differences between different wood species [5] have been reported. Mainly due to the shortage of high-quality wood from natural forest and the insufficient wood properties of plantation forest, the production and consumption of modified wood are increasing. Therefore, pyrolysis behavior of modified wood are of great importance as the amount of generated waste from modified waste will increase and part of them have a potential for energy generation through pyrolysis. So far, researchers have worked on the pyrolysis characteristics of some waste woods and wood products, such as preservative treated wood [6,7], heat-treated wood [8], formaldehyde containing wood-based panels [9]. The pyrolysis process of wood and wood products involves complex reactions, and pyrolysis characteristics of these products are greatly affected by factors such as heating rate, temperature, moisture, chemical composition and so on [10]. Yet, the pyrolysis mechanism of most modified woods is still unknown.

The combined wood modification technology of resin impregnation and heat treatment (IMPG-HT) aims at not only improving mechanical properties and dimensional stability, but also enhancing the durability and changing the color of wood to imitate that high-performance precious wood. Thus, wood modified with this combined technology has great potential to be used in the field of high-grade furniture, flooring, special furniture and building [11–15]. Previous studies on IMPG-HT wood focused on treatment technology and properties improvement of wood after modification. Whereas, thermal degradation kinetics and gases produced during pyrolysis of such modified wood has not been reported.

In this study, poplar wood lumber was modified by three different treatments, namely, heat treatment (HT), resin impregnation (IMPG), and the combined modification of resin impregnation and heat treatment (IMPG-HT). The pyrolysis behaviors of these three types of modified and untreated wood (control) were studied. The chemical compositions and pyrolysis characteristics at different heating rates of wood samples were analyzed by Fourier transform infrared spectrometry (FTIR) and thermogravimetry (TG). The pyrolysis gaseous products of the samples were analyzed by TG-FTIR. Furthermore, the activation energy was calculated by model-free methods (Flynn-Wall-Ozawa and Kissinger-Akahira-Sunose). This study is expected to improve the understanding of the pyrolysis behavior of IMPG-HT wood and to investigate its application for energy generation, and provides important information for the design of efficient pyrolysis reactors or systems.

## Materials and methods

### Materials

**Fig 1:**
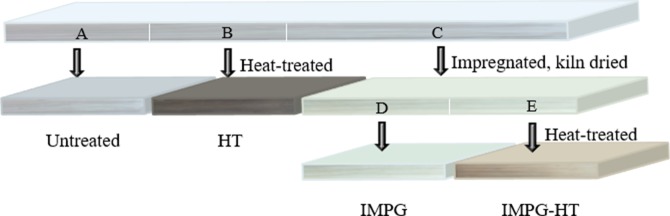
The processing diagram of poplar wood samples.

The samples were taken from 34 pieces of poplar timber (purchased from Handan, China) that are over 1300-mm-long with straight grain and no defects. The timbers were first kiln-dried until 15% moisture content(MC) and then each piece cut into three sections: Section A and B have a dimension of 20 mm× 100 mm × 320mm and C have a dimension of 20 mm × 100 mm × 640mm (radial, tangential, and longitudinal). As shown in Fig 1, section A was used as the control, whereas sections B and C were modified. The impregnated resin is a low molecular weight urea-formaldehyde (UF) resin modified with melamine and synthesized by Aimeisen Wood Processing Co. Ltd. (Handan, China). Table 1 presents the technical specifications of the impregnated resin.

**Table 1:** The technical specifications of impregnated resin.

Technical specifications	Properties
Appearance	Colorless transparent liquid
pH value	8.57
Viscosity (mPa*s(23±0.5°C))	1.5
Solid content (%)	19.56 (the solid content of original solution is about 50%)
Storage life (d)	≥40

### Wood modifications

In Fig 1, Section A was used as the control (untreated). Section B was heat-treated only to produce HT samples. Section C was first vacuum-pressure impregnated with UF resin at a pressure of 3.0 MPa for 1h, and then dried until 16% MC. The dry weight percent gain of impregnated wood varied from 16.81% to 18.11%. The impregnated section C was cross-cut into another two sections: D and E. D was used as IMPG samples, and E was subjected to HT to obtain IMPG-HT samples. Considering the energy consumption, the color change and mechanical strength loss caused by high temperature, temperature and time for HT were 180°C and 3h, respectively [12,15,16].

### Specimen preparation

Three specimens were prepared from the untreated, HT, IMPG and IMPG-HT samples, respectively. Wood pieces of 20 mm ×100 mm ×20 mm (Radial, tangential, and longitudinal) were sawn at a distance of 5 cm from the end of each specimen. The pieces were further cut into rods, ground into fine powder (100–200 meshes) with a grinder, and dried at 103°C until constant weight.

### FTIR analysis

The FTIR analysis of various samples was performed using an infrared spectrometer (Bruker VERTEX 80V, Germany) with a resolution of 2 cm^-1^ over the 4000 and 400 cm^-1^ wavenumber scanning range. Each of the dried powder samples mixed with potassium bromide powder (KBr) at a mass ratio of 1:100 were pressed into pellets for scanning. Three replicates of each powder sample were tested.

### Thermogravimetric analysis (TG) and coupled infrared spectroscopy (TG-FTIR)

TG/DTG curves of various samples were obtained by a thermogravimetric analyzer (PerkinElmer STA 8000, USA). Approximately 5.0 mg of the powder sample was added to the TG crucible and heated from 30°C to 600°C at three heating rates (10, 20 and 50°C/min) under nitrogen (99.999%, a flow of 45 mL/min) ambient. A Fourier transform infrared spectroscopy (PerkinElmer Frontier, USA) connected with the thermogravimetric analyzer (TG-FTIR) was used to obtain the infrared spectra of pyrolysis gaseous products directly from the TG experiments, where samples were heated at a rate of 20°C min^-1^ with a purge nitrogen flow of 40 mL/min. The FTIR scan range was 4000 to 500 cm^-1^ with a resolution of 2 cm^-1^ and a scan frequency of 20/min. To prevent condensation of pyrolysis gases, the temperature of gases was maintained above 200°C during the FTIR test.

### Kinetic analysis

As model-free methods can reduce the error caused by the selection of reaction models without assuming the reaction mechanism function, these methods have been increasingly used [17–19]. Flynn-Wall-Ozawa (FWO) method is a common model-free kinetic analysis method based on the integral method. Data analysis is usually performed at a specified conversion rate (*α*) on multiple TG curves measured at different heating rates, and the equation used is as follows [5,17],

$$ln\beta =ln[\frac{AE}{RG(\alpha )}]-5.331-1.052\frac{E}{RT}$$ (1)

where *β*, *T*, *A*, *R* and *E* is the heating rate (K min^-1^), the absolute temperature (K), the pre-exponential factor (min^-1^), the gas constant (8.314 kJ/mol), and the activation energy (kJ/mol). *G(α)* is an integral function of *α*, which is calculated as,

$$\alpha =\frac{m_{0}-m_{t}}{m_{0}-m_{\infty }}$$ (2)

where *m*_*0*_ (g), *m*_*t*_ (g) and *m*_*∞*_ (g) is the initial sample weight, the sample weight at time *t* (s), and the final sample weight. The plot of lnβ versus 1/*T* usually gives a straight line, and *E* at different *α* can be estimated from the slope of the fitted lines.

Kissinger-Akahira-Sunose (KAS) method is another model-free method used to calculate *E*, and the equation is expressed as follow [20,21],

$$ln\left(\frac{\beta }{T^{2}}\right)=ln[\frac{AR}{EG(\alpha )}]-\frac{E}{RT}$$ (3)

Similar to the FWO method, *E* can be graphically determined by plotting ln(*β*/*T*^2^) versus 1/*T* at different *α*.

## Results and discussions

### FTIR analysis

**Fig 2:**
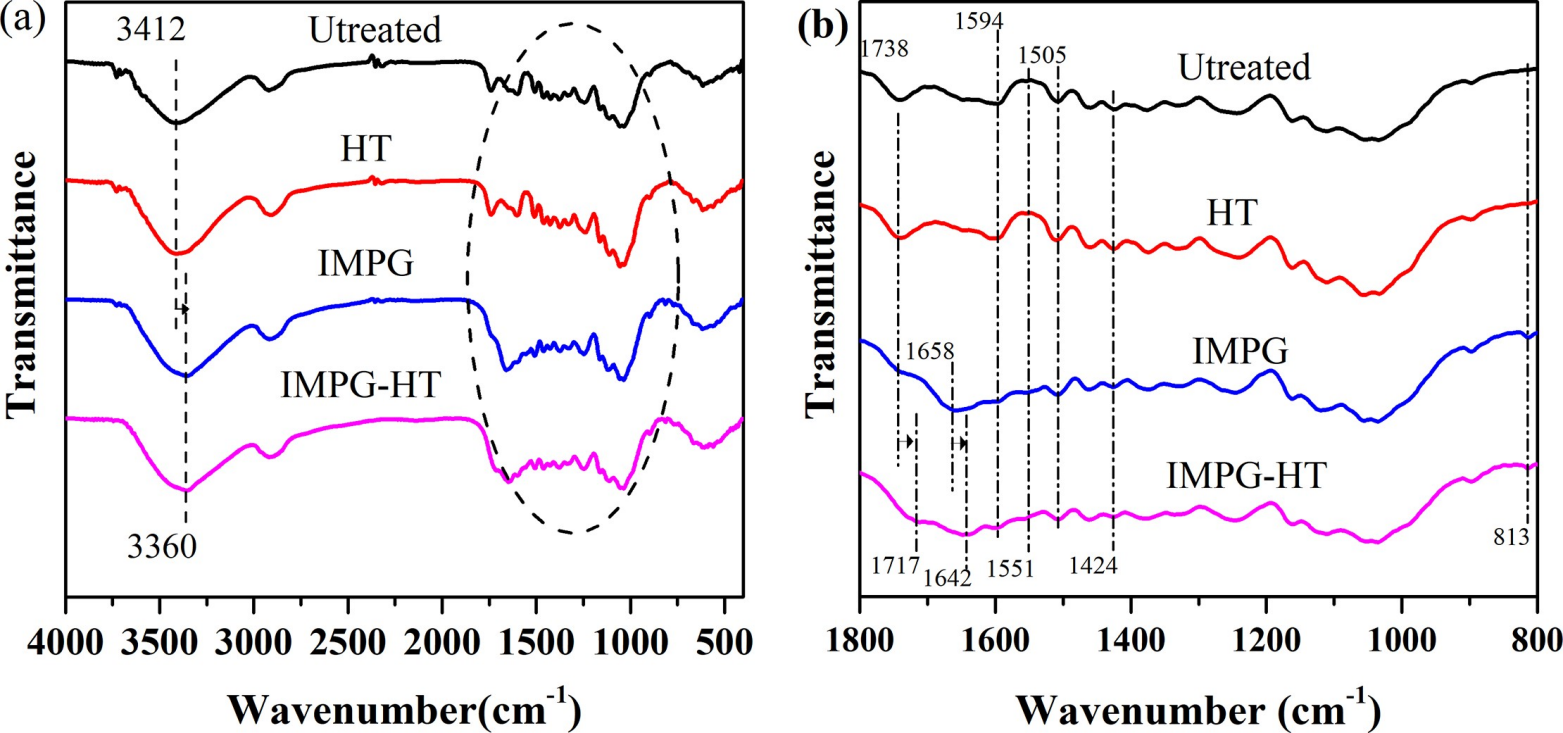
FTIR spectrum for samples. (a) FTIR spectrum in the range of 4000-400cm^-1^; (b) The amplified FTIR spectrum in the range of 1800-800cm^-1^.

Fig 2A shows the FTIR spectra of four types of samples. The fingerprint region of the wavenumber in the range of 1800–800 cm^-1^ in Fig 2A was amplified in Fig 2B to better analyze the differences among different samples. The assignment of the characteristic band [22–26] on the spectrum of the control and the relative band shifts and strength difference from the modified samples are summarized in Table 2. As shown in Fig 2, there is only a slight difference between the FTIR spectrum of HT sample and the control. The lignin characteristic peaks at 1505 cm^-1^ and 1424 cm^-1^ are slightly sharper for the HT sample, probably because of the partial degradation of hemicellulose during HT, leading to an increase of the relative content of lignin [27]. Compared with the control, the absorption peaks of O-H and N-H of IMPG and IMPG-HT samples have a redshift (shifting towards the lower wavenumber) due to the hydrogen bonding association [22,28]. Also for the IMPG and IMPG-HT samples, the characteristic peaks of UF at 1660–1640 cm^-1^ and 1551 cm^-1^ appear and the characteristic peaks of hemicellulose and lignin at 1740–1710 cm^-1^, 1594 cm^-1^, 1505 cm^-1^, and 1424 cm^-1^ weaken. Wu et al.[28] also reported this finding for poplar samples modified by the urea formaldehyde prepolymer and multilayer hot press drying. These findings suggest that the resin might have reacted with some functional groups of cell wall components. In addition, IMPG and IMPG-HT samples show N-C = N bending vibration peak at 813 cm^-1^ due to the addition of a small amount of melamine to UF. By comparing the spectra of IMPG and IMPG-HT samples, the stretching vibration peaks of C = O in hemicellulose and UF resin of IMPG wood red-shift from 1738 cm^-1^ to 1717 cm^-1^ and 1658 cm^-1^ to 1642 cm^-1^, respectively, after HT. These findings indicate that HT causes the aforementioned functional groups to form more stable intermolecular hydrogen bonds.

**Table 2:** Assignments of characteristic band on the infrared spectrum of the modified wood and their differences with untreated wood.

Wavenumbers range(cm^-1^)	Remarks	HT	IMPG	IMPG-HT
3500–3300	O-H and N-H stretching vibration	-	Redshift	Redshift
1740–1710	C = O stretching vibration of xylan acetyl groups (hemicellulose)	-	Decrease	Decrease
1660–1640	C = O stretching vibration of amide structure (UF resin)	-	Increase	Increase
1594	carboxylic acid (and carboxylate ion) group in the 4-Omethyl-d-glucuronic acid substituents (hemicellulose)s	-	Decrease	Decrease
1551	C-N stretching vibration and N-H deformation of amide structure (UF resin)	-	Increase	Increase
1505	C = C stretching of the aromatic skeleton vibrations (lignin)	Increase	Decrease	Decrease
1424	the aromatic skeleton vibrations (lignin) and CH_2_ bending deformation (cellulose)	Increase	Decrease	Decrease
813	N-C = N bending vibration (melamine)	-	Increase	Increase

### TG analysis

**Fig 3:**
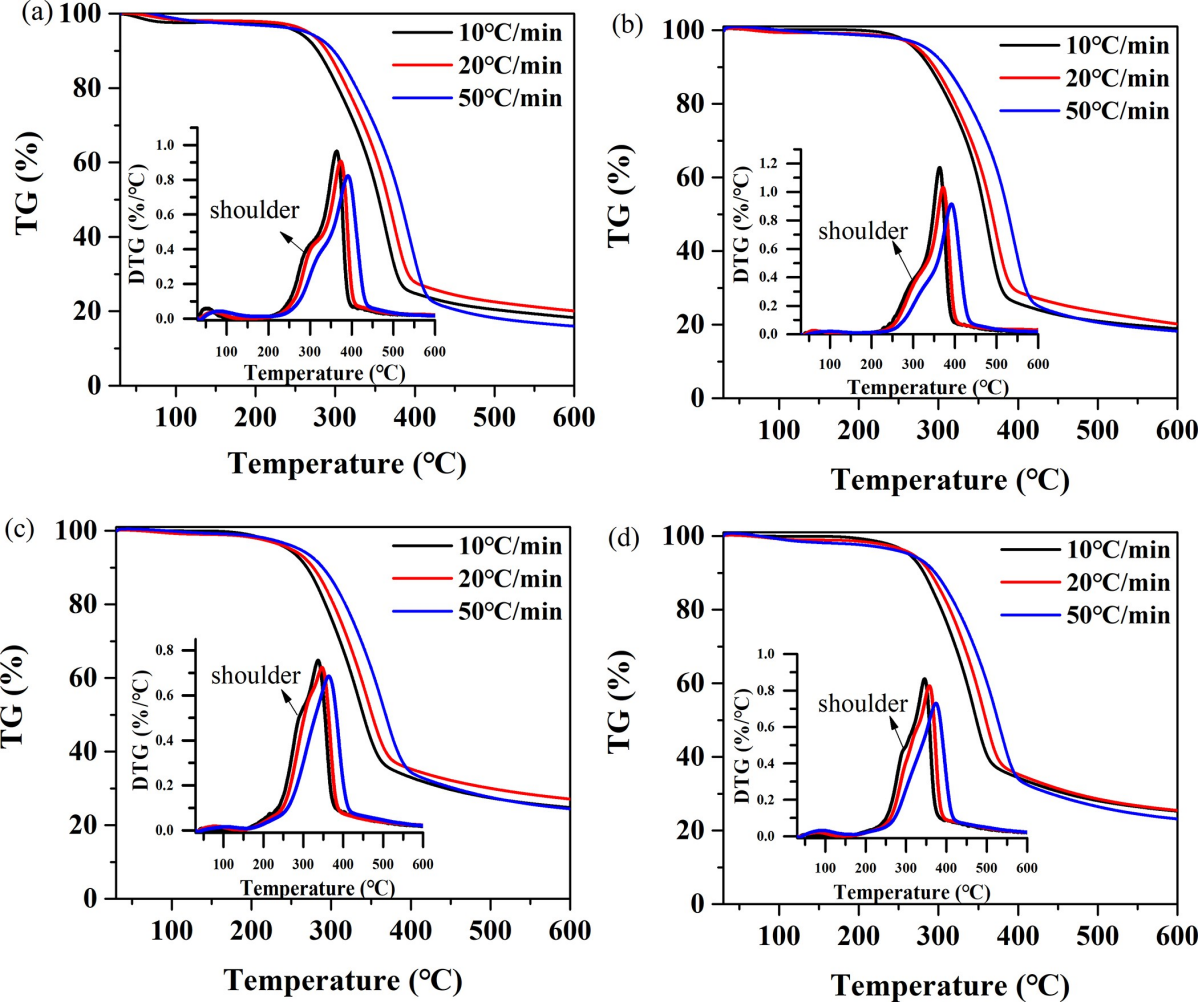
TG-DTG curves at different heating rates. (a) TG-DTG curves for Untreated samples at 10, 20 and 50 heating rates; (b) TG-DTG curves for HT samples at 10, 20 and 50 heating rates; (c) TG-DTG curves for IMPG samples at 10, 20 and 50 heating rates; (d) TG-DTG curves for IMPG-HT samples at 10, 20 and 50 heating rates.

Fig 3 shows that all the heating rates result the same trends of the pyrolysis curves for all the samples. As the heating rate increases, the TG/DTG curves shift toward higher temperature (*T*), which could be attributed to the *T* gradient difference inside and outside the sample. Zhang et al.[29] and Wang et al.[30] found the same phenomenon. Additionally, affected by the mass transfer, the maximum mass loss rate from the DTG curve decreases and the hemicellulose shoulder gradually weakens or even disappears. Because of short reaction time at a higher heating rate, the corresponding degree of reaction of the samples is lower when the same *T* is reached. Similar results were reported in previous studies [31,32]. However, heating rates have no significant effect on the total mass loss.

**Fig 4:**
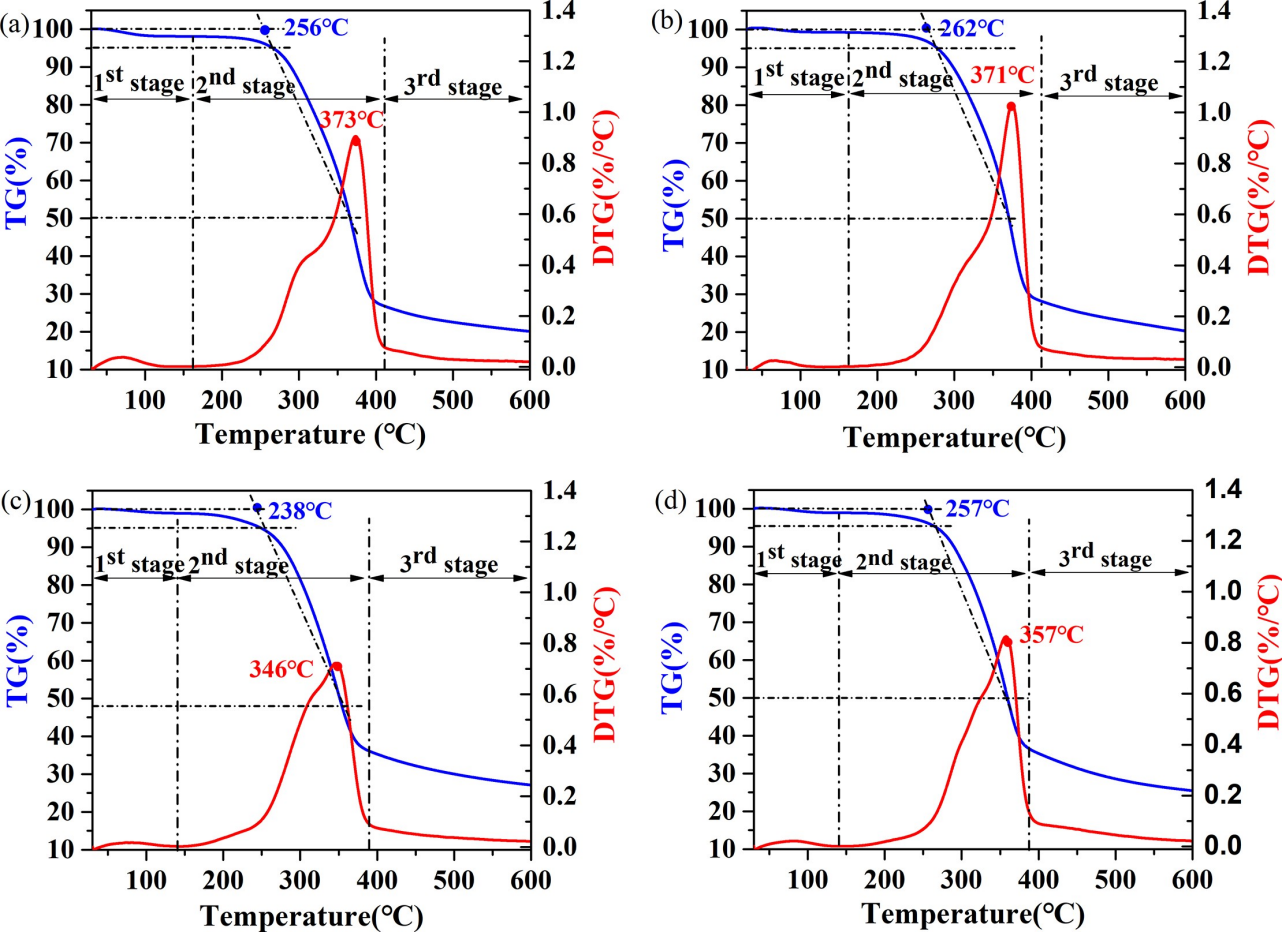
Example of TG and DTG curves. (a) TG and DTG curves for Untreated sample at a heating rate of 20°C min^-1^; (b) TG and DTG curves for HT sample a heating rate of 20°C min^-1^; (c) TG and DTG curves for IMPG sample a heating rate of 20°C min^-1^; (d) TG and DTG curves for IMPG-HT sample a heating rate of 20°C min^-1^.

Taking the heating rate of 20°C/min as an example, Fig 4 shows that pyrolysis of the samples can be divided into three main stages: the dehydration stage (1^st^ stage, <140 or 160°C), the rapid degradation stage (2^nd^ stage, 160–410°C or 140–390°C), and the slow degradation stage (3^rd^ stage, till 600°C). The 1^st^ stage is mainly caused by water evaporation and release of small molecule volatiles [33]. From the mass loss data at this stage (Table 3), the mass losses of the three modified wood samples are all lower than that of the control samples, indicating that the hygroscopicity of poplar wood decreases after the modifications [15,34,35,36]. The 2^nd^ stage is mainly caused by the degradation of hemicellulose, cellulose and resin, accompanied by partial degradation of lignin [9,37]. Table 3 shows that the mass losses of IMPG and IMPG-HT samples are significantly lower than that of the control and HT samples. This is due to the thermally unstable intermediates from the crosslinking between resin and lignin, which inhibit lignin degradation at this stage [38]. As shown in Fig 4, compared with the control, the hemicellulose shoulder (around 300°C) on the DTG curve of HT wood weakens, which is mainly due to the partial degradation of hemicellulose during HT. The shoulder of hemicellulose can hardly be observed on DTG curves of IMPG and IMPG-HT wood, which is likely caused by the crosslinking between the resin and hemicellulose during pyrolysis, leading to mass loss at the earlier stage and disappearance of the shoulder. The obtained decomposition *T* (*T*_*d*_, which was defined by American Society for Testing and Materials (ASTM)) and the DTG peak *T* (*T*_*peak*_) of HT samples are close to those of the control (Table 3). However, *T*_*d*_ and *T*_*peak*_ of IMPG samples decrease noticeably, which can be attributed to the worse thermal stability of the impregnated resin [39]. Both *T*_*o*_ and *T*_*peak*_ of IMPG-HT samples are higher than those of IMPG samples, indicating that HT is an effective means to improve the thermal stability of IMPG wood. As shown in Table 3, the maximum mass loss rate follows the order such as: HT > Control > IMPG-HT > IMPG. The reduced maximum mass loss rate of IMPG and IMPG-HT samples compared to the control, is mainly due to the expansion of resin during heating, which acts as a physical barrier preventing the heat transfer [40,41]. The maximum mass loss rates of the control and IMPG samples increase slightly after HT. Qu et al.[40] reported similar results and proposed that the increased internal space of wood, which could be caused by the partial decomposition of hemicellulose and resin during HT, promoted pyrolysis. The 3^rd^ stage mainly includes the slow carbonization of lignin and resin [29,37]. The mass losses of IMPG and IMPG-HT samples are significantly higher than those of the control and HT samples (Table 3). It is likely that the unstable intermediates formed from resin and lignin in the 2^nd^ stage further degrade into small molecules and releases at high temperatures [9]. However, the final residual masses of IMPG and IMPG-HT samples are higher than those of non-impregnated samples, and similar results were reported by Zhang et al.[29], Yorulmaz and Atimtay [42]. It can be inferred that the impregnated resin promotes coke formation.

**Table 3:** Characteristic parameters of TG and DTG curves for samples at different heating rates.

Heating rate (°C/min)	Sample	1^st^ stage		2^nd^ stage					3^rd^ stage		
		Temperature interval(°C)	Mass Loss (%)	Temperature interval(°C)	*T*_*d*_ (°C)	*T*_*peak*_ (°C)	Maximum Mass Loss rate (%/°C)	Mass Loss (%)	Temperature interval(°C)	Mass Loss (%)	Residual mass (%)
10	Untreated	30–150	2.37	150–400	252	364	0.96	72.80	400–600	6.54	18.29
	HT	30–150	0.00	150–400	260	363	1.17	75.60	400–600	5.50	18.90
	IMPG	30–120	0.05	120–380	236	337	0.75	65.19	380–600	9.95	24.82
	IMPG -HT	30–120	0.08	120–380	266	346	0.86	63.69	380–600	10.97	25.26
20	Untreated	30–160	1.90	160–410	256	373	0.91	71.25	410–600	6.76	20.09
	HT	30–160	0.76	160–410	262	371	1.03	71.12	410–600	7.94	20.18
	IMPG	30–140	1.00	150–390	238	346	0.72	62.93	390–600	8.96	27.11
	IMPG -HT	30–140	1.04	150–390	257	357	0.82	62.68	390–600	10.82	25.46
50	Untreated	30–180	2.80	180–430	251	390	0.82	75.37	430–600	5.83	16.00
	HT	30–180	1.05	180–430	275	391	0.91	74.36	430–600	6.36	18.23
	IMPG	30–150	0.73	150–420	248	363	0.68	66.85	420–600	7.97	24.45
	IMPG -HT	30–150	1.78	150–420	251	374	0.73	66.59	420–600	8.50	23.13

### Pyrolysis kinetics analysis

**Fig 5:**
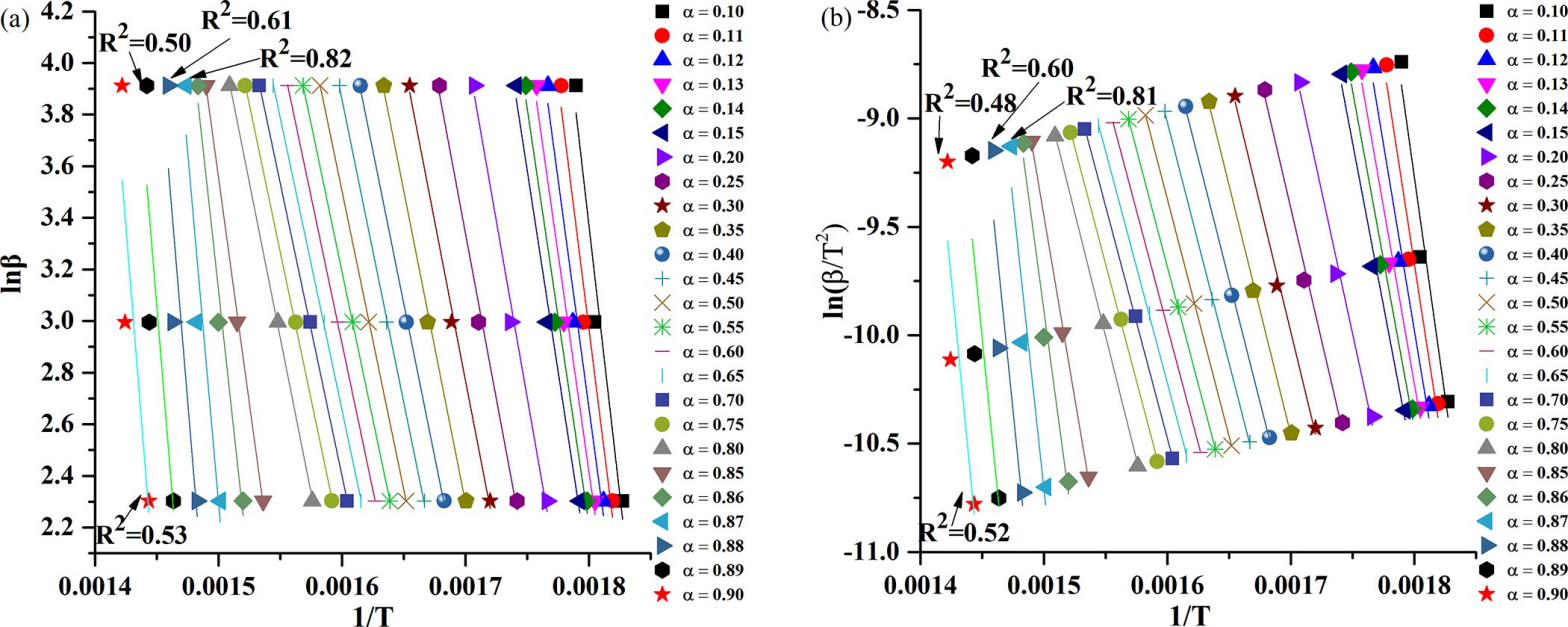
The linear fitting diagrams of IMPG-HT samples. (a) The linear fitting diagrams of IMPG-HT samples obtained by FWO method within the conversion range of 0.10<*α*<0.90; (b) The linear fitting diagrams of IMPG-HT samples obtained by KAS method within the conversion range of 0.10<*α*<0.90.

Fig 5 presents the linear fitting diagram of activation energy of IMPG-HT samples obtained by FWO and KAS methods. The fitted lines with better correlation can be used to calculate the minimum energy required for the start of pyrolysis reactions (denoted as *E*). As the correlation coefficients (*R*^*2*^) is very low when *α*>0.86 (Fig 5A and 5B), they couldn’t be accepted to calculate *E*. The *R*^*2*^ and *E* of all samples at different *α* obtained by FWO and KAS methods are summarized in S1 Table. The *E* values estimated from the two methods are nearly equivalent with a deviation of less than 3%. The activation energy of the control, HT, IMPG and IMPG-HT samples are in the range of 180-200kJ /mol, 170-200kJ /mol, 160-300kJ /mol and 180-350kJ /mol, respectively.

**Fig 6:**
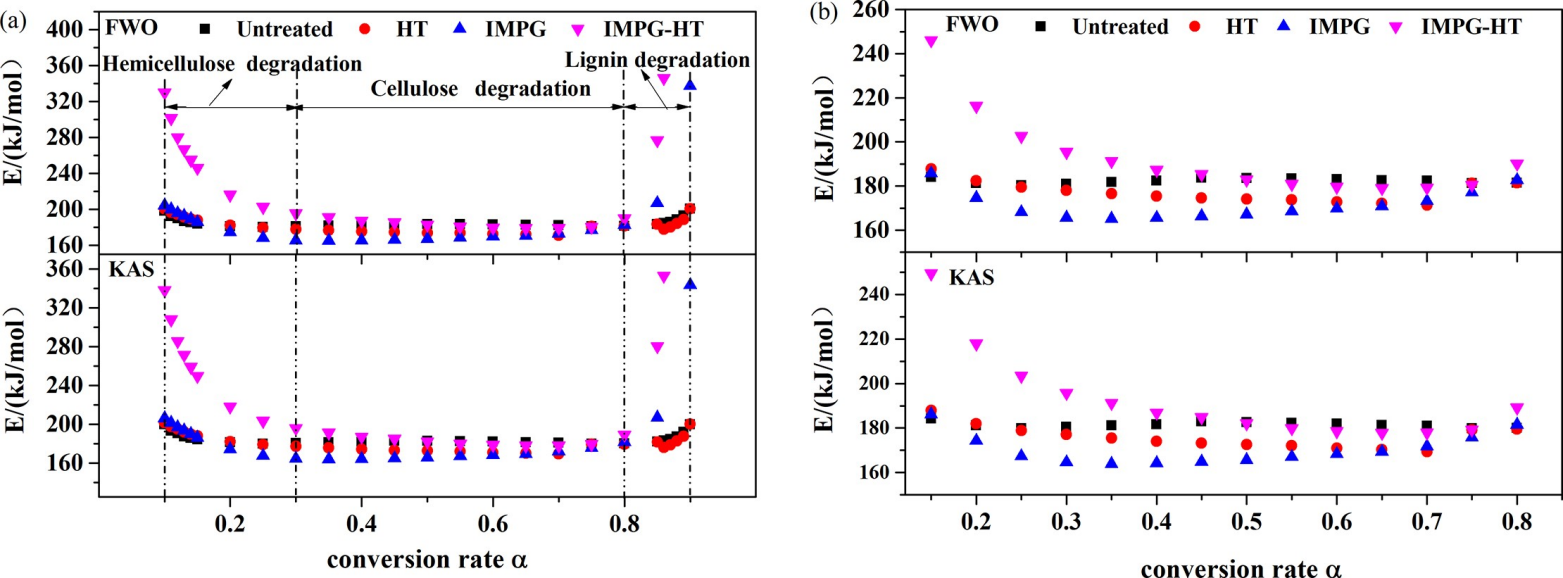
Activation energy (*E*) distributions at different conversion rate (*α*) calculated by the FWO and KAS methods. (a) *E* distributions for samples at 0.10 < *α*<0.90; (b) *E* distributions for samples at 0.15 <*α*<0.80.

The *E* distributions at different *α* calculated from FWO and KAS methods are presented in Fig 6A. The *E* distributions calculated by the two methods for various samples are similar, and all show a trend of decreasing first, then stabilizing and finally increasing. Ma et al. and Han et al. also found the similar distribution trend in wood [43] and medium-density fibreboard [44]. The decreasing trend of *E* distributions at 0.10<*α*<0.30 is mainly caused by the degradation of hemicellulose, which has a multi-branched chain structure. The trend of *E* at 0.30<*α*<0.80 is related to degradation of cellulose. The unbranched ordered polymer chain structure of cellulose leads to a relatively stable trend. The *E* distribution trend at 0.80<*α*<0.90 is mainly caused by the pyrolysis carbonization of lignin, which is responsible for the increase of low-reactive coke content and change of decomposition mechanisms [43–45]. To better compare the modified samples with the control, Fig 6B plots the distribution of *E* within the conversion rate range of 0.15–0.80. Fig 6A and 6B show that compared to the control, the HT samples have higher *E* at 0.10<*α*<0.20 and lower *E* at 0.20<*α*<0.90, indicating that HT firstly inhibits pyrolysis and then promotes it. The higher *E* in the initial stage may result from the decrease of active sites due to partial degradation of hemicellulose during HT. The lower *E* in the middle and later stages may be due to the active cellulose formed during HT, and its degradation requires lower energy [43]. Additionally, the increase in internal space is also one of the reasons for promoting pyrolysis [40].

Fig 6 shows that compared to the control, the *E* of the IMPG samples is higher at 0.10<*α*<0.15 and 0.75<*α*<0.90, but is lower at 0.15<*α*<0.75. It indicates that IMPG inhibits pyrolysis at the initial and final stages but promotes pyrolysis in between. The higher *E* at 0.10<*α*<0.15 is caused by the inhibited degradation of hemicellulose due to the alkaline environment brought in by impregnated UF resin [46]. The lower *E* at 0.15<*α*<0.75 is due to crosslinking of wood components promoted by resin in the middle stage of pyrolysis, but crosslinking also leads to more stable carbonization, which inhibits wood pyrolysis afterwards [29,46], causing higher *E* at 0.75<*α*<0.90.

Similar to IMPG’s effect on wood pyrolysis, IMPG-HT also inhibits pyrolysis at the initial and final stages, but promotes pyrolysis in between. Since a more stable chemical structure is formed during a second step HT through the reaction of resin with wood components, IMPG-HT wood has significantly higher *E* in a wider *α* range (0.10<*α*<0.45) compared to the control. Besides, the crosslinking reaction is promoted in a narrower *α* range (0.45<*α*<0.75). These results suggest that HT improves the thermal stability of IMPG wood.

### TG-FTIR analysis

**Fig 7:**
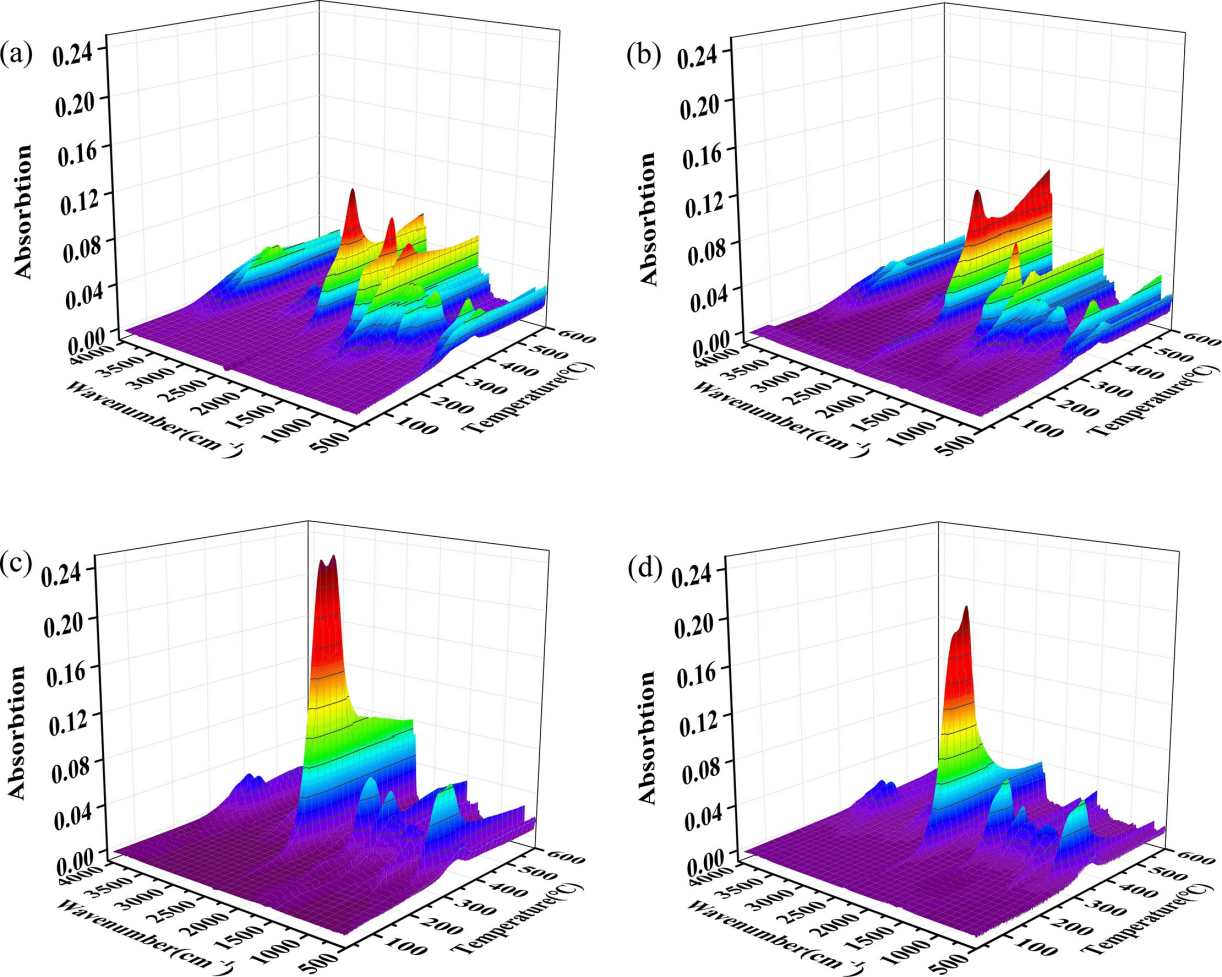
3D spectrogram of TG-FTIR of Untreated(a), HT(b), IMPG(c) and IMPG-HT(d) samples at the heating rate of 20°C min^-1^.

Fig 7 gives the 3D FTIR spectrogram of volatiles in the pyrolysis of samples at the heating rate of 20°C min^-1^, showing the variation of absorption intensity with *T* and wavenumber. Since the absorption intensity at a specific wavenumber is linearly dependent on the relative concentration of volatile components [47,48], the stronger characteristic absorption peak corresponds to the higher relative concentration of the volatile components. The gaseous products release information obtained from Fig 7 basically agrees with the results from the DTG curves. Most of the volatile components are released in the range of 160–410°C, and as the *T* increases, sharp peaks or shoulders also appears on the gases spectrum.

**Fig 8:**
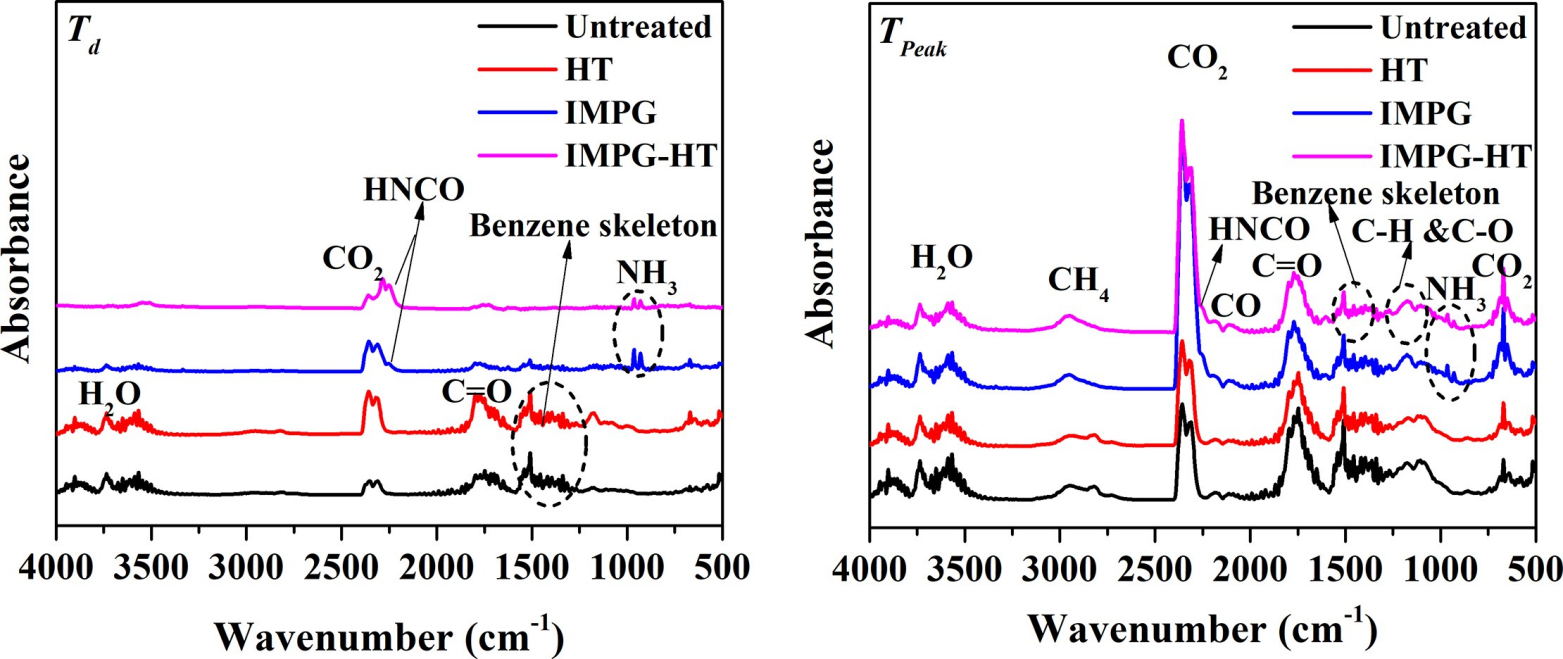
FTIR spectra of the pyrolysis gaseous products. (a) FTIR spectra of the pyrolysis gaseous products at *T*_*d*_ with a heating rate of 20°C min^-1^; (b) FTIR spectra of the pyrolysis gaseous products at *T*_*peak*_ with a heating rate of 20°C min^-1^.

The nature of volatile components can be identified from the characteristic bands on the FTIR spectra (Table 4) [3,5,20,47,49]. The spectra of the pyrolysis gaseous products of different samples at *T*_*d*_ and *T*_*peak*_ at a heating rate of 20°C min^-1^ is given in Fig 8 as an example. At *T*_*d*_, the pyrolysis gaseous products of the control and HT sample are similar, mainly including H_2_O, CO_2_, and organic components such as aldehydes, ketones, acids, aromatic hydrocarbons, alkanes, alcohols and phenols. However, the intensity of CO_2_ of HT sample is higher than that of the control. Some components of wood have been degraded to intermediate products during HT, which depolymerize to CO_2_ at the beginning of the main pyrolysis, resulting in a relatively higher concentration of CO_2_.

**Table 4:** The characteristic bands of gaseous products during pyrolysis.

Wavenumbers range(cm^-1^)	Functional groups	Vibrations	Compounds
4000–3500	O-H	stretching	H_2_O
2400–2260, 726–586	C = O	stretching, bending	CO_2_
2260–2000	C-O	stretching	CO
3000–2700	C-H, -CH_3_	stretching	CH_4_
1900–1650	C = O	stretching	Aldehydes, ketones, acids
1690–1450	C = C, benzene skeleton	stretching	Aromatics
1460–1000	C-O, C-C, C-H	stretching	Alkanes, alcohols, phenols, ethers and lipids
1000–900	N–H	out-of-plane vibration	NH_3_
2350–2200	-N = C = O	stretching	HNCO

On the contrary, the gaseous products species (mainly CO_2_, HNCO and NH_3_) of IMPG and IMPG-HT samples at *T*_*d*_ are quite different from the control. HNCO and NH_3_ are derived from the cleavage of C-N bond in UF resin [50], and almost no organic components derived from wood degradation can be observed, indicating that pyrolysis of the resin dominates the initial main pyrolysis. The NH_3_ characteristic peak of IMPG sample is more obvious than that of IMPG-HT sample. This is because some resin in the IMPG-HT samples has reacted with wood components to form a more stable nitrogen-containing chemical structure that inhibits the release of NH_3_.

Fig 8 also shows that the HT sample produces the same gaseous products species at *T*_*d*_ and *T*_*peak*_, which is similar to the control as well. However, the characteristic peak of each component produced by the HT sample is slightly weaker than that of the control. This result can be explained by the fact that some wood components have been degraded and volatilized during HT. The gases of IMPG and IMPG-HT samples are mainly CO_2_, HNCO, NH_3_, CH_4_, CO and other volatile organic components degraded from wood components. Unlike the results reported by Jiang et al.[49], no HCN is detected here, probably because the concentration of HCN is too small for the analyzer to detect accurately. The characteristic peaks of CO_2_ and CO are significantly higher than those of the control, which is mainly due to the significant contribution of C = O fracture in the resin. For both IMPG and IMPG-HT samples, the characteristic peak area of HNCO is larger than that of NH_3_, indicating that more HNCO is produced during pyrolysis, which is consistent with the results reported by Zhang et al.[29] and Girods et al.[50]. The intensity of CO_2_ of IMPG-HT sample is significantly lower than that of IMPG sample, mainly because part of the resin has been degraded into small molecules and evaporated during further HT. Similarly, the pyrolysis of IMPG-HT sample produces slightly less *N*-containing gases than that of IMPG sample.

## Conclusions

IMPG-HT changes the chemical components of poplar wood, thus changes its pyrolysis behavior. *T*_*d*_ and *T*_*peak*_ of IMPG wood are decreased than those of the control, whereas a second step HT compensates for the shortcomings of poor thermal stability of IMPG wood. The *E* calculation based on KAS and FWO methods also supports this conclusion. Additionally, both TG and *E* analysis indicate that IMPG and IMPG-HT wood promotes coke formation. The effect of resin on the pyrolysis gases of wood is mainly reflected in nitrogen-containing gases such as HNCO and NH_3_. It also shows that a second step HT decreases the relative concentration of CO_2_ and *N*-containing gas produced during pyrolysis.

## Supporting information

S1 Table The parameters for different conversion values obtained by FWO and KAS method. (DOCX)
