# Correction: Effects of impregnation combined heat treatment on the pyrolysis behavior of poplar wood

**DOI:** 10.1371/journal.pone.0236899

**Published:** 2020-07-23

**Authors:** The PLOS ONE Staff

The third author’s name is spelled incorrectly. The correct name is: Chenyang Cai.

An additional affiliation is missing for the sixth author. Jiabin Cai is also affiliated with Co-innovation Center of Efficient Processing and Utilization of Forest Resources, Nanjing, Jiangsu, China.

The publisher apologizes for the errors.

## References

[1] Wu M, Jin J, Cai C, Shi J, Xing X, Cai J (2020) Effects of impregnation combined heat treatment on the pyrolysis behavior of poplar wood. PLoS ONE 15(3): e0229907 10.1371/journal.pone.0229907 32182254 PMC7077805

