# Supplementary material for: Effects of impregnation combined heat treatment on the pyrolysis behavior of poplar wood
Source: PLoS One. 2020 Mar 17;15(3):e0229907. doi: 10.1371/journal.pone.0229907 (PMC7077805; doi:10.1371/journal.pone.0229907)
Supplement: S1 Table — (DOCX) [file pone.0229907.s001.docx]

**S1 Table. The parameters for different conversion values obtained by FWO and KAS method**

| ***α*** | **Control** | | | | **HT** | | | | **IMPG** | | | | **IMPG-HT** | | | | |
| --- | --- | --- | --- | --- | --- | --- | --- | --- | --- | --- | --- | --- | --- | --- | --- | --- | --- |
|  | **FWO** | | **KAS** | | **FWO** | | **KAS** | | **FWO** | | **KAS** | | **FWO** | | **KAS** | | |
|  | ***E***  **(kJ/mol)** | ***R^2^*** | ***E***  **(kJ/mol)** | ***R^2^*** | ***E***  **(kJ/mol)** | ***R^2^*** | ***E***  **(kJ/mol)** | ***R^2^*** | ***E***  **(kJ/mol)** | ***R^2^*** | ***E***  **(kJ/mol)** | ***R^2^*** | ***E***  **(kJ/mol)** | ***R^2^*** | ***E***  **(kJ/mol)** | ***R^2^*** |  |
| 0.10 | 198.49 | 0.9434 | 199.60 | 0.9384 | 200.66 | 0.9351 | 201.67 | 0.9290 | 204.47 | 0.9669 | 206.08 | 0.9639 | 330.05 | 0.9284 | 338.02 | 0.9247 |  |
| 0.11 | 192.31 | 0.9635 | 193.04 | 0.9601 | 196.57 | 0.9335 | 197.32 | 0.9271 | 199.95 | 0.9761 | 201.27 | 0.9739 | 301.41 | 0.9502 | 307.84 | 0.9473 |  |
| 0.12 | 189.88 | 0.9768 | 190.43 | 0.9746 | 194.47 | 0.9357 | 195.07 | 0.9294 | 195.79 | 0.9798 | 196.83 | 0.9778 | 280.09 | 0.9631 | 285.36 | 0.9608 |  |
| 0.13 | 186.83 | 0.9836 | 187.17 | 0.9820 | 191.63 | 0.9338 | 192.04 | 0.9272 | 192.77 | 0.9825 | 193.61 | 0.9807 | 266.82 | 0.9701 | 271.36 | 0.9681 |  |
| 0.14 | 185.55 | 0.9894 | 185.78 | 0.9884 | 189.61 | 0.9317 | 189.87 | 0.9247 | 189.22 | 0.9844 | 189.83 | 0.9828 | 255.04 | 0.9753 | 258.93 | 0.9736 |  |
| 0.15 | 184.07 | 0.9922 | 184.18 | 0.9915 | 187.76 | 0.9294 | 187.89 | 0.9222 | 185.79 | 0.9851 | 186.19 | 0.9835 | 246.02 | 0.9793 | 249.40 | 0.9778 |  |
| 0.20 | 181.28 | 0.9993 | 181.06 | 0.9993 | 182.34 | 0.9170 | 181.99 | 0.9080 | 174.64 | 0.9878 | 174.28 | 0.9864 | 216.21 | 0.9850 | 217.88 | 0.9838 |  |
| 0.25 | 180.31 | 0.9997 | 179.87 | 0.9997 | 179.50 | 0.9009 | 178.84 | 0.8900 | 168.16 | 0.9888 | 167.32 | 0.9874 | 202.56 | 0.9886 | 203.37 | 0.9876 |  |
| 0.30 | 180.97 | 0.9984 | 180.41 | 0.9981 | 177.96 | 0.8905 | 177.07 | 0.8782 | 165.68 | 0.9877 | 164.58 | 0.9861 | 195.39 | 0.9930 | 195.70 | 0.9923 |  |
| 0.35 | 181.79 | 0.9960 | 181.12 | 0.9955 | 176.58 | 0.8895 | 175.48 | 0.8768 | 165.15 | 0.9847 | 163.90 | 0.9827 | 191.22 | 0.9965 | 191.18 | 0.9962 |  |
| 0.40 | 182.36 | 0.9946 | 181.59 | 0.9939 | 175.38 | 0.8977 | 174.10 | 0.8856 | 165.55 | 0.9814 | 164.21 | 0.9790 | 187.20 | 0.9988 | 186.84 | 0.9987 |  |
| 0.45 | 183.65 | 0.9940 | 182.83 | 0.9932 | 174.62 | 0.9120 | 173.20 | 0.9013 | 166.30 | 0.9781 | 164.89 | 0.9752 | 185.32 | 0.9995 | 184.76 | 0.9995 |  |
| 0.50 | 183.58 | 0.9930 | 182.64 | 0.9921 | 174.10 | 0.9255 | 172.56 | 0.9163 | 167.14 | 0.9761 | 165.68 | 0.9729 | 182.80 | 0.9999 | 182.02 | 0.9999 |  |
| 0.55 | 183.34 | 0.9928 | 182.29 | 0.9918 | 173.74 | 0.9359 | 172.10 | 0.9278 | 168.53 | 0.9748 | 167.04 | 0.9715 | 180.86 | 1.0000 | 179.89 | 1.0000 |  |
| 0.60 | 183.04 | 0.9920 | 181.90 | 0.9910 | 172.72 | 0.9433 | 170.96 | 0.9360 | 169.83 | 0.9744 | 168.33 | 0.9711 | 179.59 | 0.9999 | 178.47 | 0.9999 |  |
| 0.65 | 182.58 | 0.9915 | 181.33 | 0.9904 | 172.13 | 0.9481 | 170.27 | 0.9413 | 170.80 | 0.9727 | 169.26 | 0.9691 | 178.99 | 0.9997 | 177.76 | 0.9997 |  |
| 0.70 | 182.34 | 0.9906 | 181.00 | 0.9894 | 171.26 | 0.9525 | 169.31 | 0.9461 | 173.25 | 0.9709 | 171.76 | 0.9671 | 179.14 | 0.9996 | 177.85 | 0.9996 |  |
| 0.75 | 181.27 | 0.9897 | 179.80 | 0.9883 | 181.27 | 0.9897 | 179.24 | 0.9867 | 177.15 | 0.9664 | 175.77 | 0.9621 | 180.50 | 0.9994 | 179.20 | 0.9993 |  |
| 0.80 | 181.49 | 0.9898 | 179.96 | 0.9885 | 181.49 | 0.9898 | 179.44 | 0.9872 | 182.71 | 0.9580 | 181.50 | 0.9528 | 190.05 | 0.9995 | 189.15 | 0.9994 |  |
| 0.85 | 183.53 | 0.9916 | 182.01 | 0.9904 | 183.53 | 0.9916 | 181.55 | 0.9897 | 206.93 | 0.9130 | 206.80 | 0.9039 | 276.73 | 0.9977 | 280.13 | 0.9976 |  |
| 0.86 | 184.59 | 0.9924 | 183.11 | 0.9914 | 177.87 | 0.9845 | 176.02 | 0.9824 | - | 0.8727 | - | 0.8608 | 345.94 | 0.9642 | 352.86 | 0.9620 |  |
| 0.87 | 186.00 | 0.9941 | 184.57 | 0.9933 | 180.33 | 0.9926 | 178.58 | 0.9916 | - | 0.8269 | - | 0.8132 | - | 0.8193 | - | 0.8109 |  |
| 0.88 | 188.55 | 0.9962 | 187.23 | 0.9957 | 184.27 | 0.9999 | 182.71 | 0.9998 | - | 0.8277 | - | 0.8155 | - | 0.6123 | - | 0.5972 |  |
| 0.89 | 193.02 | 0.9986 | 191.89 | 0.9984 | 188.77 | 0.9766 | 187.40 | 0.9739 | - | 0.8638 | - | 0.8548 | - | 0.4990 | - | 0.4810 |  |
| 0.90 | 200.65 | 0.9995 | 199.89 | 0.9995 | 200.65 | 0.9995 | 200.03 | 0.9976 | 337.31 | 0.9210 | 343.37 | 0.9158 | - | 0.5333 | - | 0.5162 |  |
